# Comparison in decision-making between bulimia nervosa, anorexia nervosa, and healthy women: influence of mood status and pathological eating concerns

**DOI:** 10.1186/s40337-015-0050-6

**Published:** 2015-04-02

**Authors:** Junko Matsumoto, Yoshiyuki Hirano, Noriko Numata, Daisuke Matzuzawa, Shunichi Murano, Koutaro Yokote, Masaomi Iyo, Eiji Shimizu, Michiko Nakazato

**Affiliations:** United Graduate School of Child Development, Osaka University, Kanazawa University, Hamamatsu University School of Medicine, Chiba University and University of Fukui, Suita, Japan; Research Center for Child Mental Development, Graduate School of Medicine, Chiba University, Chiba, Japan; Department of Regional Disaster Medicine, Graduate School of Medicine, Chiba University, Chiba, Japan; Tochigi Shimotsuga General Hospital, Tochigi Medical Center, Tochigi, Japan; Department of Child Psychiatry, Graduate School of Medicine, Chiba University, Chiba, Japan; Department of Cognitive Behavioral Physiology, Graduate School of Medicine, Chiba University, Chiba, Japan; Department of Clinical Cell Biology and Medicine, Graduate School of Medicine, Chiba University, Chiba, Japan; Department of Psychiatry, Graduate School of Medicine, Chiba University, Chiba, Japan; Center for Forensic Mental Health, Chiba University, Chiba, Japan

**Keywords:** Anorexia nervosa, Bulimia nervosa, Decision-making, Iowa Gambling Task, Weight-concern, Anxiety, Depressive mood

## Abstract

**Background:**

Decision-making is reported to be impaired in anorexia nervosa (AN) and bulimia nervosa (BN), but the influence of mood status, pathophysiological eating, and weight concerns on the performance of decision-making ability between AN and BN is still unclear. The aims of this study were to investigate differential impairments in the decision-making process between AN, BN, and healthy controls (HC), and secondly, to explore the role of mood status, such as anxiety, depression, pathological eating, and weight concerns, in decision-making ability.

**Methods:**

Patients suffering from AN (n = 22), BN (n = 36) and age-matched HC (n = 51) were assessed for their decision-making abilities using the Iowa Gambling Task (IGT). Self-reported questionnaires including the Eating Disorder Examination Questionnaire (EDE-Q), the Bulimia Investigatory Test, Edinburgh (BITE), the Eating Disorders Inventory, the Maudsley Obsessive-Compulsive Inventory measuring obsessive-compulsive traits, the Hospital Anxiety and Depression Scale, and the Toronto Alexithymia Scale were used to assess pathological eating concerns and attitude to feelings.

**Results:**

Significant differences in IGT performance were observed between BN and HC. Significant negative correlation was found between IGT performance and the BITE symptom subscale in AN. In BN, there was a negative correlation between the EDE-Q weight concerns subscale and IGT performance. It was also found that increased anxiety, depression, and eating/weight concerns predicted poorer decision-making.

**Conclusion:**

Different patterns of association between pathological eating concerns/behaviors and performances in decision-making ability were found between AN, BN, and HC. Anxiety, depressive mood status, and eating/weight concerns were related to decision-making ability.

## Background

Eating disorders (EDs) are severe and enduring psychiatric disorders of eating behavior, including extreme, unhealthy decreases in food intake as well as severe overeating, accompanied by feelings of distress or excessive concern about body shape or weight [1]. Three types of eating disorders are recognized by the text revision of the fourth edition of the Diagnostic and Statistical Manual of Mental Disorders (DSM-IV-TR) [2]: anorexia nervosa (AN), bulimia nervosa (BN), and eating disorder not otherwise specified (EDNOS).

Decision-making is affected by the combination of emotional representations, sensitivity to immediate reward and long-term outcome according to the somatic marker hypothesis (SMH) [3-5]. Multiple cognitive functions such as attention, memory, learning [6,7], risk-taking, and obsessive-compulsive traits [8] have been suggested as being involved in performances in decision-making [9,10]. A previous study by Tchanturia et al. found impairment of emotional signal by skin conductance (SCR), showing the lowest emotional signal by demonstrating a lack of ability to be aware of emotional signal during decision-making task [11]. In BN, a previous study showed no significant correlation between SCR and performance in decision-making [12]. For this reason, it is unclear whether the AN and BN groups have different deficits in emotional skills during decision-making.

The role of emotion, specifically anxiety or worry, may influence the decision-making process [13]. High levels of worry may have expected consequences of future events that influence the performances of decision-making [14]. The majority of people with EDs have high levels of anxiety [15], worry, a defining cognitive feature, and a maintenance factor of anticipatory anxiety [16]. Heightened anxiety levels may affect the process of decision-making in AN or BN patients. However, few studies have focused on the effect of mood status on a prospect during the decision-making process in AN compared with BN. Two studies showed that decision-making in patients with AN may be related to anxiety [17,18], whereas other studies showed that they were unrelated [7,19]. Some studies suggested significant associations between measures of depressive disorder and decision-making [20,21], but a number of other studies have indicated that depressive symptoms did not significantly influence decision-making ability in patients with EDs [7,12,22-24].

Alexithymia is commonly described as consisting of four features: (1) difficulty identifying and describing subjective feelings; (2) difficulty distinguishing between feelings and the bodily sensations of emotional arousal; (3) lack of fantasy; and (4) an externally orientated cognitive style [25]. Previous studies demonstrated that patients with EDs use maladaptive eating behaviors (e.g., binging, purging, or dietary restriction) as a way to avoid or cope with their emotions [26,27], with many clinical studies suggesting that eating disorder symptoms are associated with emotional dysfunction [28,29], with clear functional links expressed between emotional states and both bulimic and restrictive pathology. It has been suggested that patients with EDs tend to show alexithymia, but only one study has examined the relation in EDs in comparison with healthy controls (HC), showing that alexithymia was unrelated to decision-making in AN [30].

The Iowa Gambling Task (IGT) is a neuropsychological task that tests the decision-making ability to sacrifice immediate rewards in order to achieve long-term gain [3]. IGT assesses set-shifting ability, reaction to reward and punishment, and learning ability to decide advantageous over disadvantageous choice under uncertainty [31]. IGT is underpinned by SMH, a theory that, in essence, posits that decision-making under uncertainties is guided by emotional responses to anticipated positive and negative consequences [5,6]. Neuroimaging findings suggest that activation of the mesolimbic pathway during wins and decreased activation of the inferior frontal gyrus during losses lead to repeated selections in reward and punishment in IGT [32]. IGT was developed for functional assessment, given that patients with ventromedial prefrontal cortex (vmPFC) and limbic system dysfunction show severe impairments in decision-making.

Increasing evidence suggests neuropsychological traits such as poor set-shifting ability [19], weak central coherence [19,33], a dysfunction of the reward circuit, including a preference for immediate reward despite long-term adverse consequences [15], higher sensitivity to punishment [34], and poor insight into illness [35,36] in AN. In previous studies, memory function [37], skin conductance response [11], body mass index (BMI) [38], anxious mood [18] and impaired decision-making ability were indicated in AN. Regarding the domain of decision-making ability, several studies have reported that individuals with AN show impaired decision-making ability as reflected by poorer performance on IGT [11,22,39-41].

In BN, decision-making ability was impaired in some studies [12,22,41], showing that obsessive-compulsive traits [42] and pathological eating symptoms may be related to impaired decision-making ability, which in turn may lead to real-life risk-taking and immediate reward-seeking behavior such as binge eating and purging.

Thus, in total, relatively few studies have been conducted concerning the decision-making ability between AN, BN and HC [12,37,43,44]. In addition, it has remained unclear whether the performances of decision-making are distinguished by pathological eating concerns/behaviors, mood status (anxiety, depression), and attitude to feelings such as alexithymia between AN, BN, and HC.

The hypotheses of this study were: 1) decision-making performances can be distinguished in AN, BN and HC, and 2) decision-making deficits are related to mood status such as anxiety, depression, alexithymia, and pathological eating symptoms.

The aims of this study were to investigate differential impairments in the decision-making process between AN, BN, and HC, and secondly, to explore the role of mood status such as anxiety, depression, attitudes to feelings, and pathological weight concerns in decision-making ability.

## Methods

### Participants

The patients of this study were 58 females recruited from Chiba University Hospital, Japan (22 AN; 36 BN). They were interviewed by a senior psychiatrist assessing criteria for AN and BN as defined by DSM-IV [2]. In addition, the M.I.N.I. International Neuropsychiatric Interview translated into Japanese (M.I.N.I.) [45] was applied. Exclusion criteria for patients with AN and BN were a history of brain injury, epilepsy, psychosis or drug dependence. The AN group included restrictive (n = 9) and binge eating/purging (n = 13) subtypes. The BN group included purging (n = 34) and non-purging (n = 2) subtypes. A total of 7 females (2 AN and 5 BN) had the following comorbidities: dysthymia (5%; 3 with BN), panic disorder (2%; 1 with BN), somatoform disorder (2%; 1 with AN), anxiety disorder (2%; 1 with AN), and alcohol dependence (2%; 1 with BN). Seventeen percent of all patients were taking serotonergic drugs (SSRIs) (Table 1).

**Table 1:** **Demographic and clinical characteristics of anorexia nervosa patients (AN), bulimia nervosa patients (BN), and healthy controls (HC)**

	**Eating disorders**				**Healthy control**				
	**AN (n = 22)**		**BN (n = 36)**		**HC (n = 51)**				
**Observed mean, M(SD)**	**Mean**	**SD**	**Mean**	**SD**	**Mean**	**SD**	***F-value***	***p-value***	***Post hoc***
Age (years)	25.77	6.26	25.94	5.81	23.82	5.58	1.71	0.19	n.s.
Education (years)	13.23	2.20	14.07	1.87	14.00	0.91	1.24	0.30	n.s.
Duration of illness (only AN, BN)	7.24	6.47	7.15	5.80	−	−	0.00	0.96	n.s.
BMI (kg/m^2^)	15.87	2.62	19.76	2.38	20.99	1.71	76.83	***0.00***	HC>AN, HC>BN, BN>AN
TAS-20	60.07	7.95	64.00	8.16	49.50	9.71	26.89	***0.00***	AN>HC, BN>HC
HADSa	11.06	4.29	12.00	3.87	4.61	3.39	42.55	***0.00***	AN>HC, BN>HC
HADSd	9.18	4.79	11.32	4.43	3.37	3.14	41.38	***0.00***	AN>HC, BN>HC
EDE-Qg	3.23	1.60	3.97	1.26	1.07	0.89	59.89	***0.00***	AN>HC, BN>HC
EDE-Qr	3.00	1.89	3.17	1.62	0.68	0.81	39.78	***0.00***	AN>HC, BN>HC
EDE-Qe	3.39	1.70	3.72	1.63	0.47	0.64	75.27	***0.00***	AN>HC, BN>HC
EDE-Qw	3.74	1.32	4.32	1.45	1.44	1.29	44.73	***0.00***	AN>HC, BN>HC
EDE-Qs	3.96	1.23	4.56	1.23	1.70	1.25	50.96	***0.00***	AN>HC, BN>HC
BITEss	8.75	6.32	11.57	5.56	1.37	1.18	67.76	***0.00***	AN>HC, BN>HC
BITEsas	17.63	10.14	22.61	4.38	5.33	4.46	104.27	***0.00***	AN>HC, BN>HC
EDI-2	117.06	43.93	139.83	37.19	60.64	29.77	52.29	***0.00***	AN>HC, BN>HC
MOCI	10.47	5.93	13.55	6.21	7.17	3.34	15.83	***0.00***	BN>HC
**Comobidities; n=**									
Dysthymia	−		3						
Panic disorder	−		1						
Somatoform	1		−						
Anxiety disorder	1		−						
Alcohol dependence	−		1						
**Medication; n=**									
SSRIs	−		10						

BMI: body mass index; TAS-20: Toronto Alexithymia Scale; EDE-Qg: Eating Disorder Examination Questionnaire (global score); EDE-Qr: restricting; EDE-Qe: eating concern; EDE-Qw: weight concern; EDE-Qs: shape concern; HADSa: Hospital Anxiety and Depression Scale (anxiety); HADSd: Hospital Anxiety and Depression Scale (depression); BITEsas: Bulimia Investigatory Test, Edinburgh: (symptom scale); BITEss: Bulimia Investigatory Test, Edinburgh (severity scale); MOCI: Maudsley Obsessive-Compulsive Inventory; EDI-2: Eating Disorders Inventory 2; SSRIs: selective serotonin reuptake inhibitors.

In bold: *p*-value <0.05, n.s.: not significant.

HC (n = 51) were recruited through local advertisements and a website from a potential pool of university students and volunteers. Age-matched HC underwent an interview by a senior psychiatrist using M.I.N.I. [45], and they were determined to have no family history of psychiatric conditions, history of brain injury, epilepsy, psychosis, current substance abuse or dependence, risk of suicide, mental retardation, autistic spectrum disorders, comorbid depression and bipolar disorders, and that their BMI (body mass index) was between 19 and 25 kg/m^2^.

### Procedures

All participants, female native Japanese speakers, were between age 18 and 38 (mean = 24.92, SD = 5.83 years). After the study had been described to the participants, their written informed consent was obtained. The ethics committee of the Chiba University Graduate School of Medicine approved the study protocol.

### Measurements

### Instruments

#### Toronto Alexithymia Scale (TAS-20)

The Toronto Alexithymia Scale [46,47], Japanese version with established validity and reliability [48], is a 20-item self report questionnaire measuring alexithymia. It includes three subscales: difficulty identifying feelings, difficulty describing feelings, and externally oriented (concrete) thinking. Cut-off scores for TAS-20 are equal to or less than 51 for non-alexithymic individuals, and equal to or greater than 61 for alexithymia. Scores of 52–60 indicate possible alexithymia.

#### Hospital Anxiety and Depression Scale (HADS)

The Hospital Anxiety and Depression Scale [49], Japanese version established as valid and reliable [50], is a widely used self-report scale developed to detect states of depression, anxiety and emotional distress among patients being treated for a variety of clinical problems. The scale consists of eight questions assessing depression (HADS-d) and eight assessing anxiety (HADS-a). The optimal cut-off point is said to be greater than or equal to 8 for the identification of suspicious cases and greater than or equal to 11 for safe cases on both subscales [49].

#### Eating Disorder Examination Questionnaire (EDE-Q)

The Eating Disorders Examination Questionnaire [35], Japanese version, which was established for its validity as well as reliability [51], is a widely used 36-item self-report questionnaire that assesses the eating disorders-related level of symptoms over the past 28 days. EDE-Q generates two types of data. First, 22 scaled items plus one unscaled item (items 1–15 and 29–36) provide subscale scores reflecting the severity of aspects of the ED psychopathology. Second, 13 more items (items 16–28) provide data on six key behavioral features of ED in terms of presence/absence and frequency with which the behavior occurred, and loss of control. EDE-Q includes four subscale scores, Restricting (EDE-Qr), Eating concern (EDE-Qe), Shape concern (EDE-Qs) and Weight Concern (EDE-Qw), which are included in this assessment, the response format of which is a 7-point Likert-type scale (0: never; 6: every day). The subscale scores are obtained by calculating the average of the items forming each subscale, and the global score (EDE-Qg) is the average of the four subscale scores.

#### Bulimia Investigatory Test, Edinburgh (BITE)

The Bulimia Investigatory Test, Edinburgh [52,53], Japanese version, recognized for its validity and reliability [54], is a 33-item self-report measure designed to identify individuals with symptoms of bulimia or binge eating. BITE consists of two subscales: the symptom scale (BITE-sas), which measures the degree of symptoms present, and the severity scale (BITE-ss), which provides an index of the severity of binging and purging behavior as defined by their frequency.

#### Eating Disorders Inventory-2 (EDI-2)

The Eating Disorder Inventory-2 contains 91 items and is a self-report questionnaire designed for use with those aged 12 years or older. This measure assesses features commonly associated with anorexia nervosa and bulimia nervosa but does not provide diagnoses for eating disorders [55]. EDI-2 consists of 11 subscales including bulimia, body dissatisfaction, drive for thinness, perfectionism, ineffectiveness, interpersonal distrust, interoceptive awareness, maturity fears, asceticism, impulse regulation and social insecurity. The Japanese version of EDI-2, which has been established as valid and reliable [56], was used to assess the presence of eating disorders.

#### Maudsley Obsessive-Compulsive Inventory (MOCI)

The Maudsley Obsessive-Compulsive Inventory [57], Japanese version, recognized as being valid and reliable [58], is a true-false format self-report questionnaire developed for evaluating obsessive-compulsive symptoms to discriminate obsessive patients from other neurotic patients and nonclinical individuals. The test is composed of 30 dichotomous items, such that the total score for a participant will range between 0 (absence of symptoms) and 30 (maximum presence of symptoms).

The levels of psychopathology in eating disorders were measured using the scores of BITE, total scores of EDI-2, and EDE-Q subscores.

### Neuropsychological assessment

#### Iowa gambling task [3,59]

Decision-making ability of participants was evaluated using IGT, which is a neuropsychological task based on emotion-guided evaluation. Participants are required to choose one card at a time from four available decks of cards (100 trials) in this task. The goal of the task is to win as much money as possible. To accomplish a task, participants have to detect, from a long-term perspective, which are the most advantageous decks. First, participants were given both the task instructions and 200,000 Japanese yen (approximately US$1,666) of play money. Each time participants choose a card, they will win some money; however, on turning over each card they also will, seldom or sometimes, have to pay a penalty according to a pre-programmed schedule of reward and punishment. Gains and losses differ for each card selected from the four decks. Decks A and B are “bad decks (disadvantageous)”, and the other decks, C and D, are “good decks (advantageous)”, because, in the former, while participants receive 10,000 Japanese yen (approximately US$83), the losses are also higher, such that these decks cost more in the long run. In contrast, the latter will lead to overall gains in the long run (receiving less money, but punishments are also smaller). The 100 choices were divided into five blocks of 20 choices each. We calculated the number of advantageous cards (decks C and D) selected in total.

### Statistical analyses

All statistical analyses were performed using SPSS 21.0 (IBM Corp., Armonk, NY). Demographic and clinical variables for ED and HC groups were compared using one-way analyses of variance (ANOVAs).

IGT scores were defined as the number of choices from the advantageous decks (C and D) minus the number of choices from the disadvantageous decks (A and B) for all 100 trials. This net score (decks[C + D] - decks[A + B]) calculated for each 20-choice time block enables the assessment of learning during the task. A total net score for the 100 selections is also calculated. A score of <10 was established by Bechara et al. as the threshold for deficit of decision-making on IGT, given the maximum net score achieved by vmPFC patients was <10 [9]. A 5 × 3 repeated-measures ANOVA was carried out with the net scores of the five blocks [C + D]-[A + B](1–20, 21–40, 41–60, 61–80, 81–100) as the repeated-measures variable and the three diagnostic groups (AN, BN, and HC) as a between-subjects variable. Effect size was calculated using Cohen’s *d*, with *d* = 0.2 regarded as a small effect, *d* = 0.5 as a medium effect, and *d* = 0.8 as a large effect [60]. Pearson’s correlations were used to examine the relationship between IGT performance and demographic and clinical variables in the whole sample and in each group, respectively. Finally, multiple regression analysis was performed for all participants to detect the best predictors of IGT performance, using IGT performance as the dependent variable and all questionnaire scores and subscale scores showing significant relationships as independent variables. In all analyses, the statistical significance level was set at *p* < 0.05 (2-tailed tests).

## Results

### Sample characteristics

Demographic and clinical characteristics are summarized in Table 1. The three groups did not differ in terms of age (*F* (2,106) = 1.71; *p* =0.19) and education (*F* (2, 65) = 1.24; *p* = 0.30). In addition, no significant difference between the patient groups in terms of illness duration was found (*F* (1, 52) = 0.003; *p* < 0.096). On the other hand, significant differences were obtained for BMI and clinical self-report measures (TAS-20, EDE-Q, HADS, BITE, MOCI, and EDI-2). *Post hoc t* tests revealed that AN and/or BN differed from HC for most of the dimensional assessments, while no significant differences were found with respect to the overall questionnaires between AN and BN.

### Decision-making performances

#### Group comparisons in IGT total net scores [C + D]-[A + B](1–100 choices)

Results from IGT are presented in Table 2. The prevalence of decision-making impairment (IGT < 10, [9]) was approximately 45% in AN, 44% in BN patients, and 45% in HC. No significant group differences were found in the mean IGT total net scores (*F* (2,103) = 1.06; *p* = 0.35), indicating that the decision-making abilities of the three groups were quite similar.

**Table 2:** **Decision-making ability on the Iowa Gambling Task (IGT) in AN, BN, and HC**

	**Eating disorders**				**Healthy control**				
	**AN (n=22)**		**BN (n=36)**		**HC (n=51)**				
**IGT**	**Mean**	**SD**	**Mean**	**SD**	**Mean**	**SD**	***F-value***	***p-value***	***post hoc***
Block 1	-1.43	7.46	-2.00	6.01	-2.61	5.29	0.31	0.73	n.s.
Block 2	2.19	7.45	2.18	7.21	-0.31	6.96	1.62	0.20	n.s.
Block 3	3.14	7.47	1.82	7.68	4.14	7.85	0.92	0.40	n.s.
Block 4	3.90	7.50	3.15	8.82	6.49	7.44	2.03	0.14	n.s.
Block 5	2.67	10.11	2.29	8.73	7.35	9.29	3.73	***0.03***	BN<HC
Total net scores	10.48	25.53	7.50	27.09	15.06	20.99	1.05	0.35	n.s.

In bold: p-value < 0.05, n.s.: not significant.

#### Group comparisons in IGT block net scores [C + D]-[A + B](1–20, 21–40, 41–60, 61–80, 81–100)

**Figure 1:**
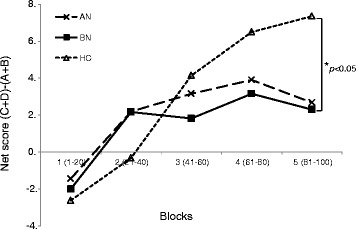
**Strategy of Iowa Gambling Task, as total number of “Advantageous” minus “Disadvantageous” cards selected in each block of 20 cards; anorexia nervosa (AN), bulimia nervosa (BN), and healthy control (HC). A significant difference between BN and HC was indicated (** ***p*** **< 0.05).**

Figure 1 shows the mean IGT scores for the three groups over the five blocks of 20 trials each. A 5 (IGT block) × 3 (group) repeated measures ANOVA was performed on net scores for all five blocks. Mauchly’s test indicated that the assumption of sphericity had been violated (χ^2^ (9) = 51.51, *p* < 0.0001), and therefore the degrees of freedom were corrected using Greenhouse-Geisser estimates of sphericity (ε = 0.78). There was no significant main effect of group (*F* (2, 103) = 1.06, *p* = 0.35, *ηp*2 = 0.02), but there was a significant main effect of block (*F* (3.14, 57.48) = 14.53, *p* < 0.0001, *ηp2* = 0.12), and a significant group × block interaction (*F* (6.28, 57.48) = 2.63, *p* = 0.02, *ηp2* = 0.05) over the IGT blocks. In the HC group, IGT performance showed a gradual increase across blocks. There was a significant task-related learning effect, as performance improved during the task for BN and HC (BN: *F* (4, 32) = 2.69; *p* = 0.04; HC: *F* (4, 47) = 15.24; *p* < 0.0001). A post-hoc least significant difference test indicated that there was a significant difference between the BN and HC groups in the final block [C + D]-[A + B](81–100), that is, performance in BN was significantly worse than in HC (*p* = 0.02). On the other hand, although no significant difference was observed between AN and HC in the final block [C + D]-[A + B](81–100), performance in AN was marginally deficient compared to HC (*p* = 0.054). The two clinical groups were not significantly different from each other in any other block. Effect sizes for between-groups differences in IGT net scores were measured using Cohen’s *d* (block 1: AN vs. HC, *d* = 0.20; BN vs. HC, *d* = 0.11; block 2: AN vs. HC, *d* = 0.36; BN vs. HC, *d* = 0.36; block 3: AN vs. HC, *d* = 0.13; BN vs. HC, *d* = 0.30; block 4: AN vs. HC, *d* = 0.35; BN vs. HC, *d* = 0.42; block 5: AN vs. HC, *d* = 0.50; BN vs. HC, *d* = 0.56).

### Group comparisons controlling for covariates

We demonstrated the same group comparisons analysis, controlling for the use of SSRIs in the IGT block net scores [C + D]-[A + B](1–20, 21–40, 41–60, 61–80, 81–100). We not only detected remaining significant differences in the IGT net scores in the final [C + D]-[A + B](81–100) choices (*p* = 0.01), but also found significant differences between BN and HC in the fourth [C + D]-[A + B](61–80) choices (*p* = 0.01).

### Association between decision-making and clinical variables

#### Correlation analysis

**Figure 2:**
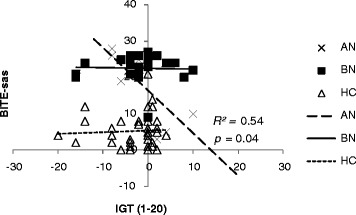
**Scatter plot shows the scores of the first block on IGT (1–20 within 100 trials) and the bulimia investigatory test, edinburgh symptom subscale (BITE-sas) for AN, BN, and HC. Negative correlation was found in AN (** ***r*** **= −0.73;** ***p*** **= 0.04).**

**Figure 3:**
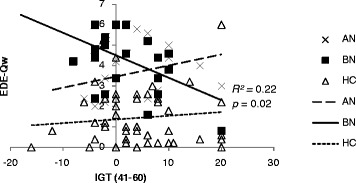
**Scatter plot shows the scores of the third block on IGT (41–60 within 100 trials) and the eating disorder examination questionnaire weight concern subscale (EDE-Qw) for AN, BN, and HC. Negative correlation was found in BN (** ***r*** **= −0.47;** ***p*** **= 0.02).**

We explored correlations among clinical measures including all scores such as TAS-20, EDE-Q, BITE, EDI-2, HADS, MOCI and IGT performance (both IGT total net scores: [C + D]-[A + B](1–100 choices) and block net scores: [C + D]-[A + B](1–20, 21–40, 41–60, 61–80, 81–100) ) in AN and BN females, respectively. Performance in the first block [C + D]-[A + B](1–20) of the IGT was negatively associated with BITE-sas in the AN group (*r* = −0.73, *p* = 0.04) (Figure 2). In the BN group, as shown in Figure 3, the IGT performance in the third block [C + D]-[A + B](41–60) was also negatively correlated with EDEQ-w (*r* = −0.47, *p* = 0.02). Therefore, we detected different patterns of association between pathological eating concerns/behaviors and the performances of decision-making ability between AN and BN.

#### Regression analysis

Multiple regression analysis was performed for all participants using demographic and clinical scores such as TAS-20, EDE-Q (four subscales: restricting, eating concern, shape concern, weight concern), HADS (depression and anxiety), EDI-2, MOCI, and BITE (symptoms and severity) as independent variables and IGT net scores [C + D]-[A + B] (1–20, 21–40, 41–60, 61–80, 81–100) as dependent variables. As shown in Table 3, the analyses revealed seven predictive factors for the third block of IGT [C + D]-[A + B](41–60): years of education (*β* = 0.77, *p* = 0.0001), EDEQ-r (*β* = 1.58, *p* = 0.0001), HADS-a (*β* = −0.69, *p* = 0.01), HADS-d (*β* = 1.44, *p* = 0.0001), EDI-2 (*β* = −0.81, *p* = 0.01), BITE-ss (*β* = −0.51, *p* = 0.03), and BITE-sas (*β* = −1.80, *p* = 0.0001). In contrast, no significant predictive factor was highlighted for the AN and BN groups, suggesting that mood status (anxiety or depression), in addition to the pathological eating/weight concerns for the prospect of decision-making were detected.

**Table 3:** **Multiple regression analysis with Iowa Gambling Task net scores (third block 41-60 within 100 trials) as the dependent variable in all participants**

**Variables**	**Beta**	***t***	***p***
education (years)	0.77	4.40	***0.00***
EDE-Qr	1.58	4.45	***0.00***
HADS (anxiety)	-0.69	-2.69	***0.01***
HADS (depression)	1.44	4.65	***0.00***
BITEss	-0.51	-2.33	***0.03***
BITEsas	-1.8	-5.01	***0.00***
EDI-2	-0.81	-2.62	***0.01***

n = 109; R^2^ = 0.556; adjusted R^2^ = 0.388; SE of estimate = 6.036.

Results showed seven variables predicting performance on the IGT.

SE: standard error; EDE-Qr: Eating Disorder Examination Questionnaire restricting subscale; HADS: Hospital Anxiety and Depression Scale; BITE: Bulimia Investigatory Test, Edinburgh; EDI-2: Eating Disorder Inventory-2.

## Discussion

In the present study, we found different profiles in IGT performance between BN, AN, and HC. As shown in Figure 1, a comparison of the performance curves of the three groups revealed that the individuals with AN and BN, as opposed to HC, failed to learn advantageous decision-making until the end of the task. Although no significant difference between AN and HC was observed, a difference between BN and HC (BN < HC, *p* = 0.02) was detected in the final block [C + D]-[A + B](81–100). Regarding total net scores, the prevalence of decision-making impairment (IGT < 10) was reported to be approximately 61% in AN and 77% in BN by Brogan et al. [44], but our data showed lower percentages. Secondly, only in the BN group, there was a significant negative correlation between the weight concern subscales and the performances of decision-making ability. These findings may be strongly confirmed by the fact that in the BN group, pathological weight concern affected the impaired decision-making ability.

A previous study has reported that patients with BN were significantly different from the HC group in blocks 3 [C + D]-[A + B](41–60) and 4 [C + D]-[A + B](61–80) [44]. In contrast, we found that BN made fewer advantageous choices than HC in the final block [C + D]-[A + B](81–100) of the task. This would suggest that pathological concerns affect ignored long-term negative consequences, which may have led to impaired decision-making ability in the final block [C + D]-[A + B](81–100) in the current study. There is a striking resemblance between the IGT performance of the patients and their real-life pathological behaviors, in which they have a tendency to reduce their food intake and/or refuse to eat, or in contrast to this pattern, repetitively overeating and purging, ignoring long-term negative consequences. In a previous study, BN subjects failed to learn an advantageous decision-making strategy by choosing immediate rewards (high gains) despite the long-term negative consequences (loss of money) as compared to HC, showing that sensitivity to gains affect these findings [23], results consistent with the current study. Boeka and Lokken [22] suggested that there are links between decision-making, weight, and eating concerns/restricting behavior in BN, and thus the authors argued that the severity of bulimic symptoms as measured by the Bulimia Test-Revised [61] and the severity of EDE-Q (restraint, eating concerns and weight concerns) contribute to decision-making ability. These data were consistent with the findings in the BN group in the current study. Brand et al. suggested that performance in decision-making was related to executive functioning but not to other neuropsychological functions, personality, or disease-specific variables in the BN group [41]. Regarding the task, in comparison to HC, the patients with BN tended to choose disadvantageous alternatives more frequently, possibly due to a tendency to fail to learn from the anterior half of the task, which might be linked to real-life pathological behaviors.

On the other hand, although performance in AN was marginally deficient compared to HC (*p* = 0.05) in the last trial [C + D]-[A + B](81–100) of the IGT, the current study does not support results from other studies [11,39,40], showing that AN patients failed to reach a significant difference in decision-making compared to HC. One explanation for this is the small sample size of the current study. Additionally, the fewer comorbidities (AN, 2 with comorbidities; BN, 5 comorbidities) in AN might have led to better decision-making compared to BN. Interestingly, in the AN group, there was a significant correlation between bulimic symptomatology measured by the BITE symptom subscale, which measures the degree of present symptoms, and the poor performance of IGT in the first block [C + D]-[A + B](1–20). 59.1% (13/22) of the AN group had binge eating/purging subtype, which may have affected the poor performance of IGT. Thus, our first hypothesis, that the AN and BN groups present a different pattern in decision-making ability, was confirmed.

The second aim of this study was to explore the links between decision-making ability and mood status, weight/eating concerns of pathological symptoms. Using multiple regression analysis, we found that EDI-2 and BITE-ss measures predicted decision-making. These data are in line with previous investigations concerning this subject [18,22].

Both the states of anxiety and depressive mood were found to be predictors of better decision-making. These data suggest that emotional states may impact decision-making in EDs [11] as well as in HC [62-64]. Zeeck et al. reported that the urge to eat is significantly higher under negative-emotional states; negative emotions such as sadness or disappointment correlated significantly with the number of binges, whereas positive emotions did not [65]. Thereby, the ‘Network Theory of Affect’ [66], that is, affective nodes (central units), can be semantic (with straightforward meaning) or affective (with emotional meaning), which may confirm the findings of the previous study. One recent study of binge eating disorders was in line with this view, proposing that the emotional state may have a direct experience that is similar to its emotion [67].

Alexithymia, as measured by TAS-20, did not affect decision-making ability in the current study, although a higher level of alexithymia compared with HC was observed. Miyake et al. reported that there was no correlation with decision-making ability using emotional decision-making task and alexithymia in EDs [30], a result consistent with the finding of our study.

In the current study, controlling for the use of SSRIs as covariance, we detected significant difference in IGT performances between BN and HC in the fourth block [C + D]-[A + B](61–80) and the final block [C + D]-[A + B](81–100), which suggested the influence of the serotonin system in decision-making. In the previous study by Tchanturia et al. [11], 44% of AN patients were taking SSRIs, but no difference between medicated and non-medicated patients was found. Emerging data have suggested that dysregulation of serotonin circuits in cortical and limbic structures are related to anxiety, eating behaviors and body image symptoms [68]. Alterations of this system may influence mood status and decision-making process in EDs, which may lead to insights into potential treatment approaches. The question of whether cognitive impairment in EDs is an endophenotype and risk factor or whether it is a correlate of illness remains unclear from the findings in the current study. It may be suggested that the relationship of symptomatology and emotional functioning to decision-making performance improves with recovery of illness.

There are some limitations to this study. First, it should be noted that a single task such as IGT is limited in examining decision-making impairments comprehensively, and this is true for the other clinical scales as well. Second, the results are generalizable for females only, and the sample sizes were not large enough compared to previous studies [12,37,43,44], indicating that a replication with a larger group that includes males is desirable. Finally, other variables such as impulsivity, central coherence, set-shifting, and inhibitory control-confounding factors associated with decision-making deficits or emotional dysregulation should be considered in the future.

Future study should evaluate how some different emotional variables such as sadness and fear may influence the decision-making process in patients with EDs. A longitudinal study is required to investigate changes in decision-making ability in accordance with emotional states and recovery of symptomatology of illness.

## Conclusions

In conclusion, we found different profiles in IGT performance between BN, AN, and HC. Different patterns of association between pathological eating concerns/behaviors and the performances of decision-making ability were found between AN, BN, and HC. Individuals with BN, compared to HC, have a different processing pattern of decision-making ability that may be linked to pathological eating/weight concerns. Anxiety, depressive mood status, and pathological eating/weight concerns are linked to decision-making ability.

## Abbreviations

AN: Anorexia nervosa
ANOVA: One-way analyses of variance
BITE: Bulimia Investigatory Test, Edinburgh
BITEsas: Bulimia Investigatory Test, Edinburgh symptom scale
BITEss: Bulimia Investigatory Test, Edinburgh severity scale
BMI: Body Mass Index
BN: Bulimia nervosa
DSM-IV-TR: Diagnostic and Statistical Manual of Mental Disorders
EDs: Eating disorders
EDE-Q: Eating Disorder Examination Questionnaire
EDE-Qe: Eating Disorder Examination Questionnaire eating concern
EDE-Qg: Eating Disorder Examination Questionnaire global score
EDE-Qr: Eating Disorder Examination Questionnaire restricting
EDE-Qs: Eating Disorder Examination Questionnaire shape concern
EDE-Qw: Eating Disorder Examination Questionnaire weight concern
EDI-2: Eating Disorders Inventory
HADS: Hospital Anxiety and Depression Scale
HADS-a: Hospital Anxiety and Depression Scale anxiety
HADS-d: Hospital Anxiety and Depression Scale depression
HC: Healthy controls
IGT: Iowa Gambling Task
MOCI: Maudsley Obsessive-Compulsive Inventory
SMH: Somatic Marker Hypothesis
SSRI: selective serotonin reuptake inhibitor
TAS-20: Toronto Alexithymia Scale
vmPFC: Ventromedial prefrontal cortex
